# Prognostic impact of immune gene expression signature and tumor infiltrating immune cells in localized clear cell renal cell carcinoma

**DOI:** 10.1186/s40425-019-0621-1

**Published:** 2019-05-28

**Authors:** Pooja Ghatalia, Jennifer Gordetsky, Fengshen Kuo, Essel Dulaimi, Kathy Q. Cai, Karthik Devarajan, Sejong Bae, Gurudatta Naik, Timothy A. Chan, Robert Uzzo, A. Ari Hakimi, Guru Sonpavde, Elizabeth Plimack

**Affiliations:** 1 Fox Chase Cancer Center, 333 Cottman Ave, Philadelphia, PA 19111 USA; 2 University of Alabama at Birmingham, 1802 6th Ave S, Birmingham, AL 35233 USA; 3 Memorial Sloan Kettering Cancer Center, 1275 York Avenue, New York, NY 10065 USA; 4 Dana Farber Cancer Institute, 450 Brookline Ave, Boston, MA 02215 USA

**Keywords:** Immunotherapy, Tumor infiltrating lymphocytes, Renal cell carcinoma, Localized, Immune cell

## Abstract

**Background:**

The tumor immune microenvironment has become the focus of research in clear cell renal cell carcinoma (ccRCC) due to its important role in immune surveillance post nephrectomy. This study investigates the correlation of tumor infiltrating immune cell characteristics with rates of recurrence following surgery in localized ccRCC.

**Methods:**

We morphologically identified and scored tumor infiltrating lymphocytes (TILs) in hematoxylin and eosin (H&E) stained slides of patients with localized ccRCC (stage ≥T1b excluding stage IV). The University of Alabama at Birmingham (UAB) dataset (*n* = 159) was used to discover and the Fox Chase Cancer Center (FCCC) dataset (*n* = 198) was used to validate the results of morphologic immune cell analysis. We then performed gene expression analysis using the Immune Profile panel by NanoString in the UAB cohort and identified immune cells and pathways associated with recurrence, followed by validation in the Cancer Genome Atlas (TCGA) ccRCC dataset. Infiltrating immune cell types were identified by gene expression deconvolution.

**Results:**

The presence of TILs identified by morphology correlated with higher T cell, Th1, CD8+ T and Treg gene signatures. Recurrence was associated with lower T cells and higher neutrophils. Higher Teffector (Teff)/Treg ratio correlated with lower rate of recurrence and was validated in the TCGA dataset. Genes associated with adaptive immune response were downregulated in tumors that recurred. Unsupervised hierarchical clustering identified a subset of patients with over-expression of adaptive response genes including *CD8, CD3, GZMA/B, PRF1, IDO1, CTLA4, PDL1, ICOS* and *TIGIT.* These patients had higher morphologic lymphocyte infiltration and T cell gene expression. Higher levels of TILs identified by morphology correlated with higher rates of recurrence in our discovery dataset but not in our validation set.

**Conclusions:**

Recurrence of ccRCC following surgery was associated with lower T cell infiltrate, lower adaptive immune response and higher neutrophil gene expression. Presence of higher Teff/Treg ratio correlated with lower recurrence.

**Electronic supplementary material:**

The online version of this article (10.1186/s40425-019-0621-1) contains supplementary material, which is available to authorized users.

## Background

Approximately 30–40% of patients with localized clear cell renal cell carcinoma (ccRCC) develop metastatic recurrence during follow-up after surgical resection. To date, no systemic adjuvant therapy has improved overall survival despite multiple large trials [1–5]. With immune checkpoint inhibitors demonstrating improved outcomes in the setting of metastatic ccRCC [6], the potential benefit from these agents is being evaluated in the perioperative setting in ongoing studies. Therefore, there is great interest in understanding the immune microenvironment of localized ccRCC as it could have prognostic and predictive implications, and may also help patient selection for adjuvant therapy.

Studies describing the prognostic impact of intra-tumoral immune cell infiltrates (lymphocytes, plasma cells, macrophages, neutrophils) on recurrence in patients with localized renal cell carcinoma (RCC) have produced conflicting results. For example, Choueiri and Remark et al. reported that the presence of increased CD8+ T cells was associated with shorter survival in metastatic RCC patients [7, 8]. However, another study reported that the presence of CD8+ T cells is associated with better survival in localized RCC [9]. Separately, Bromwich et al. indicated that the presence of CD4+ but not CD8+ T-cell infiltrate is associated with poor survival [10]. Giraldo et al. reported that the localization of dendritic cells in the tumor microenvironment modulates the clinical impact of CD8+ T cells in ccRCC [11]. In a retrospective analysis of the S-TRAC trial using adjuvant sunitinib in high risk RCC patients, higher tumor infiltration of CD8+ T cells was associated with longer disease-free survival in the sunitinib arm [1].

To understand the role of TILs in localized ccRCC, we first morphologically identified and quantified immune cells in resected primary tumors of patients with localized ccRCC. Secondly, to identify the immune cell types and immune pathways associated with recurrence, we performed gene expression analysis of tumor samples including any infiltrate using the NanoString Immune Profile panel platform on tumors from the UAB dataset. For the NanoString analysis we used the Cancer Genome Atlas (TCGA) data for validation.

## Methods

### Patient selection

We employed a discovery sample set from the University of Alabama at Birmingham (UAB) and separate validation sample set from Fox Chase Cancer Center (FCCC) for morphologic TIL evaluation. We identified patients with ccRCC (stage T1b and above, excluding metastatic disease at presentation) who underwent surgical resection [nephrectomy or partial nephrectomy] at UAB between 2000 and 2013, had baseline clinical and pathology data as well as documentation objective tumor recurrence available with a minimum follow-up of 2 years. Patients undergoing surgery at FCCC for localized ccRCC (2009–2012) were used as the validation dataset, which employed similar selection criteria for stage and follow-up. For validation of the gene expression analysis, similar selection criteria were employed to select appropriate patients from the TCGA database.

### Central pathologic review

Identical methods were used for central pathology review by UAB and FCCC pathologists blinded to clinical outcomes. Pathologic scoring of immune infiltrates included lymphocytes/plasma cells, neutrophils and macrophages. All available hematoxylin and eosin (H&E) stained archival slides from a given tumor were evaluated. An average of 6 slides containing tumor were reviewed per case. Immune cells within the tumor and at the tumor margin were considered for grading. Immune cells outside the tumor were excluded. We also excluded immune cells in tumor zones with crush artifacts, necrosis or hemorrhage. The lymphocyte/plasma cell infiltration was given a score of 0, 1, 2, 3 or 4 based on the maximum number of foci identified in any H&E slide of a given patient’s tumor block. For example, if 2 foci were identified in an H&E slide of one block and 3 foci were identified in another block of the same tumor, then a score of “3” was given. When 4 or more foci were identified, a score of 4 was given. Due to the paucity of neutrophils and hemosiderin laden macrophages, they were scored as present or absent. Of note, the morphologic review of H&E stained slides does not distinguish between various subtypes of lymphocytes or mononuclear cells and scoring is subjective.

### NanoString platform for gene expression analysis

Tumor tissue slides from UAB samples with clear cell histology and the highest immune infiltration were demarcated for histologic macrodissection performed on 10 μm sections. RNA was isolated from dissected tumor tissue using RNeasy FFPE kit (Qiagen, Valencia, CA). A tissue surface area of approximately 50 mm^2^ was used to harvest the necessary amount of RNA (~ 50 ng). RNA integrity was assessed via the 260/280 ratio using nanodrop. RNA was input directly into the nCounterTM platform (NanoString Technologies, Seattle, WA) for the hybridization reaction containing color-coded molecular barcodes representing 770 Immune Profile Panel genes, including 40 housekeeping genes.

A codeset specific to a 100-base region of the target mRNA was custom designed by NanoString Technologies using a 3′ biotinylated capture probe and a 5′ reporter probe tagged with a specific fluorescent barcode, creating two sequence-specific probes for each target transcript. Probes were hybridized to 100 ng of total RNA for 19 h at 65 °C and then applied to the nCounterTM preparation station for automated removal of excess probe by immobilization of probe-transcript complexes on a streptavidin-coated cartridge.

### Data processing

Data were collected using the nCounter™ Digital Analyzer by counting the individual barcodes. Each codeset included probes for the 770 immune related genes, spiked-in external RNA consortium positive and negative controls, including 40 housekeeping genes. Background hybridization was determined using spiked-in negative controls. All signals below mean background plus 2 standard deviations were considered to be below the limits of detection and set to mean background. A normalization factor was calculated from the spiked-in exogenous positive controls in each sample and applied to the raw counts from the nCounter™ output data.

### TCGA ccRCC cohort RNA-Seq analysis

For the TCGA clear cell cohort (KIRC), FASTQ files were downloaded from GDC and aligned against the hg19 assembly by STAR. Gene level count values were then computed by the summarizeOverlaps function from the R package “GenomicAlignments” with UCSC hg19 KnownGene as the base gene model. The Union counting mode was used and only mapped paired reads were considered. FPKM (Fragments Per Kilobase Million) values were then computed from gene level counts by using fpkm function from the R package “DESeq2”.

### Immune infiltration deconvolution analysis

The nCounter PanCancer Immune Profiling Panel gene annotation data, which includes 24 immune cell type (aDC, B-cell, CD8 T-cell, Cytotoxic cell, DC, Eosinophils, iDC, Macrophages, Mast cell, Neutrophils, NK CD56bright cell, NK CD56dim cell, NK cell, pDC, T helper cell, T-cell, Tcm, Tem, TFH, Tgd, Th1 cell, Th17 cell, Th2 cell, and Treg) gene signatures and 4 immune response category (Adaptive, Innate, Inflammation and Humoral) gene signatures, were downloaded from NanoString products site (https://www.NanoString.com/products/gene-expression-panels/hallmarks-cancer-gene-expression-panel-collection/pancancer-immune-profiling-panel?jumpto=SUPPORT). For the UAB cohort, the NanoString expression values were log2 transformed and followed by quantile normalization. For the KIRC TCGA cohort, the RNA-Seq FPKM expression values were log2 transformed for analysis. The immune cell type scores and immune response category scores were then calculated by taking the mean of the normalized/transformed expression values of genes defined in the corresponding NanoString gene signature (log2 mean). In addition to NanoString gene signatures, the ESTIMATE [12] algorithm was also employed in assessing the overall immune infiltration (ImmuneScore), stromal content (StromalScore), and the combined (ESTIMATEScore) score of the samples. We also derived a the immune cytolytic score (‘CYT’) based on the geometric mean of TPM (Transcripts Per Kilobase Million) transcript levels (0.01 offset) of two key cytolytic effectors, granzyme A (GZMA) and perforin (PRF1), according to the work of Hacohen et al. [13] and the Teff score according to the work of McDermott et al. [14] The average expression of all genes in the Teff signature was computed as the Teff score for the UAB. For KIRC TCGA cohort, the average of log2 transformed FPKM value of signature genes was computed as the Teff score. To derive the Teff / Treg ratio for UAB cohort, the Teff score is divided by the Treg score, For KIRC TCGA cohort, the anti-log2 values of Teff and Treg scores were used for deriving Teff / Treg ratio.

### Differentially expressed genes (DEG) analysis

The R package “limma” (version 3.29.0) [15] was used for DEG. Limma powers differential expression analyses for RNA-sequencing and microarray studies. Limma returned empirical Bayes moderated-t *p*-values and adjusted *P*-values (Q-value) to correct for multiple comparisons testing using the Benjamini-Hochberg method to control the false discovery rate (FDR). Genes with a FDR less than 0.3 and fold change greater than 1.5 times were reported.

### Immunohistochemistry

Immunohistochemical staining was carried out according to standard methods on both the UAB and FCCC samples. Briefly, 5- μm FFPE sections were deparaffinized and hydrated. Sections were then subjected to heat-induced epitope retrieval with 0.01 M citrate buffer (pH 6.0). Endogenous peroxidases were quenched by the immersion of slides in 3% hydrogen peroxide solution. The sections were incubated overnight with primary antibodies to CD3 (Rabbit, Ready to use, Ventana 790–4341) CD8a (Rabbit, Ready to use, Ventana M7103) and FoxP3 (Rabbit, 1:30 dilution, Cell signaling 98,377) at 4 °C in a humidified slide chamber. Immonodetection was performed using the Dako Envision+ polymer system and immunostaining was visualized with the chromogen 3, 3′-diaminobenzidine.

The sections were then washed, counterstained with hematoxylin, dehydrated with ethanol series, cleared in xylene, and mounted. As a negative control, the primary antibody was replaced with normal mouse/rabbit IgG to confirm absence of specific staining. All slides were viewed with a Nikon Eclipse 50i microscope and photomicrographs were taken with an attached Nikon DS-Fi1 camera (Melville, NY, USA). Immunostained slides were also scanned using an Aperio ScanScope CS 5 slide scanner (Aperio, Vista, CA, USA). Scanned images were then viewed and captured with Aperio’s image viewer software (ImageScope, version 11.1.2.760, Aperio).

### Statistical analysis

#### Correlation between recurrence and morphologic immune cells

Chi-square tests were used to explore the association between recurrence and morphologic TILs. Univariate and multivariate logistic regressions were performed to determine the odds of tumor recurrence adjusting for clinical pathological factors. Multicollinearity among variables was assessed using variance inflation factors (VIF) value of 5. Results were considered statistically significant at the 0.05 level. Analysis was performed using SAS v.9.4 (SAS Institute Inc., Cary, North Carolina).

#### Correlation between recurrence and NanoString cell types in UAB dataset and TCGA KIRC dataset

To test the association between immune phenotypes and recurrence, the non-parametric test, Wilcoxon rank-sum test, was used to test the immune phenotype score difference between recurrent and non-recurrent samples. The difference of the means of standardized immune infiltration deconvolution scores (Z scores) between recurrent and non-recurrent samples was calculated.

#### Unsupervised heirarchical clustering

NanoString expression values were converted into gene-wise standardized values (Z scores) and the corresponding genes from 4 immune response categories defined by the NanoString were extracted from the matrix and used for non-supervised hierarchical clustering in UAB cohort along with sample clinical information and immune infiltration level of certain cell types as annotation tracks by using the R package Pheatmap. Any common genes among immune response categories were excluded from clustering.

## Results

### Patient population

**Fig. 1:**
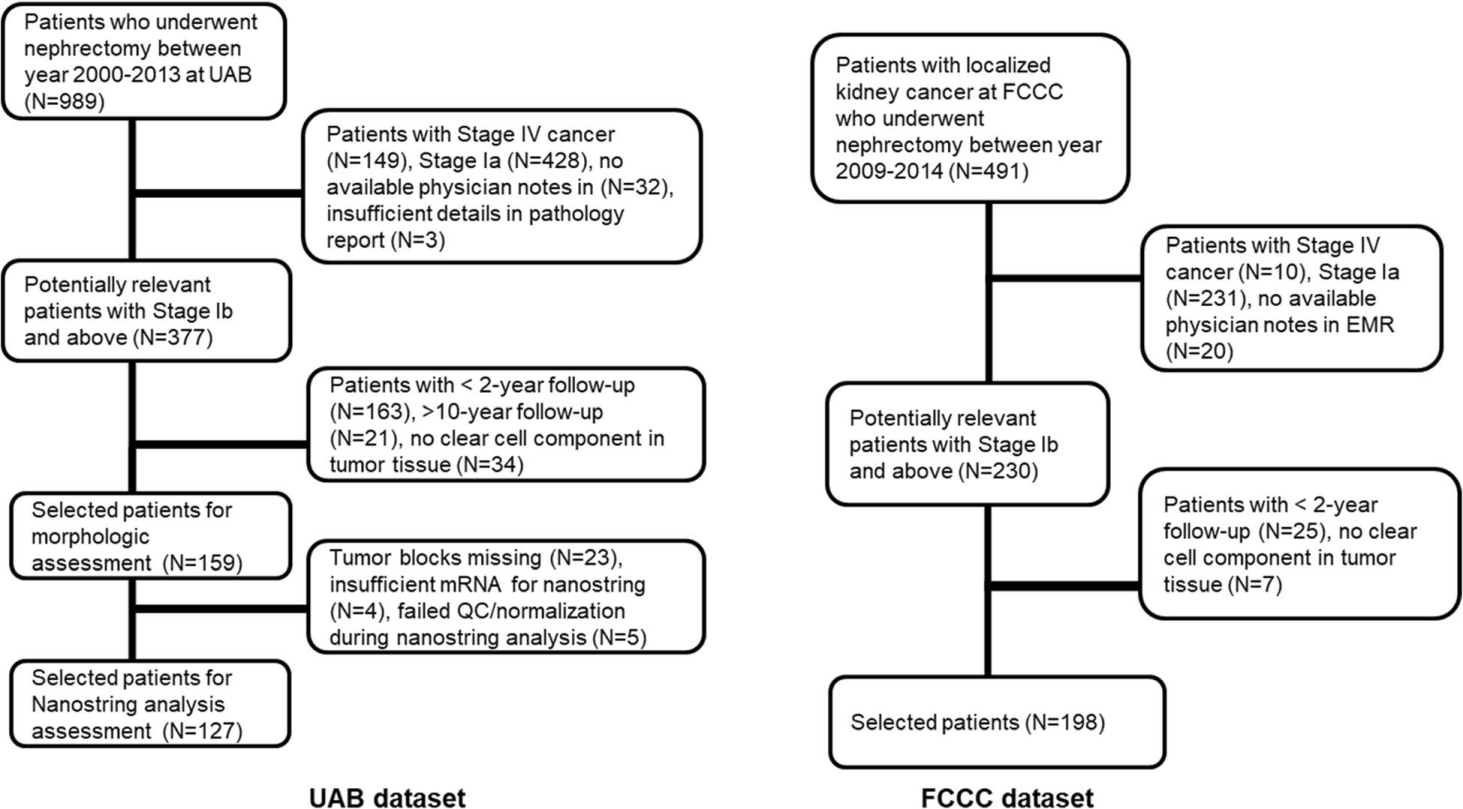
Patient selection for UAB and FCCC datasets

**Fig. 2:**
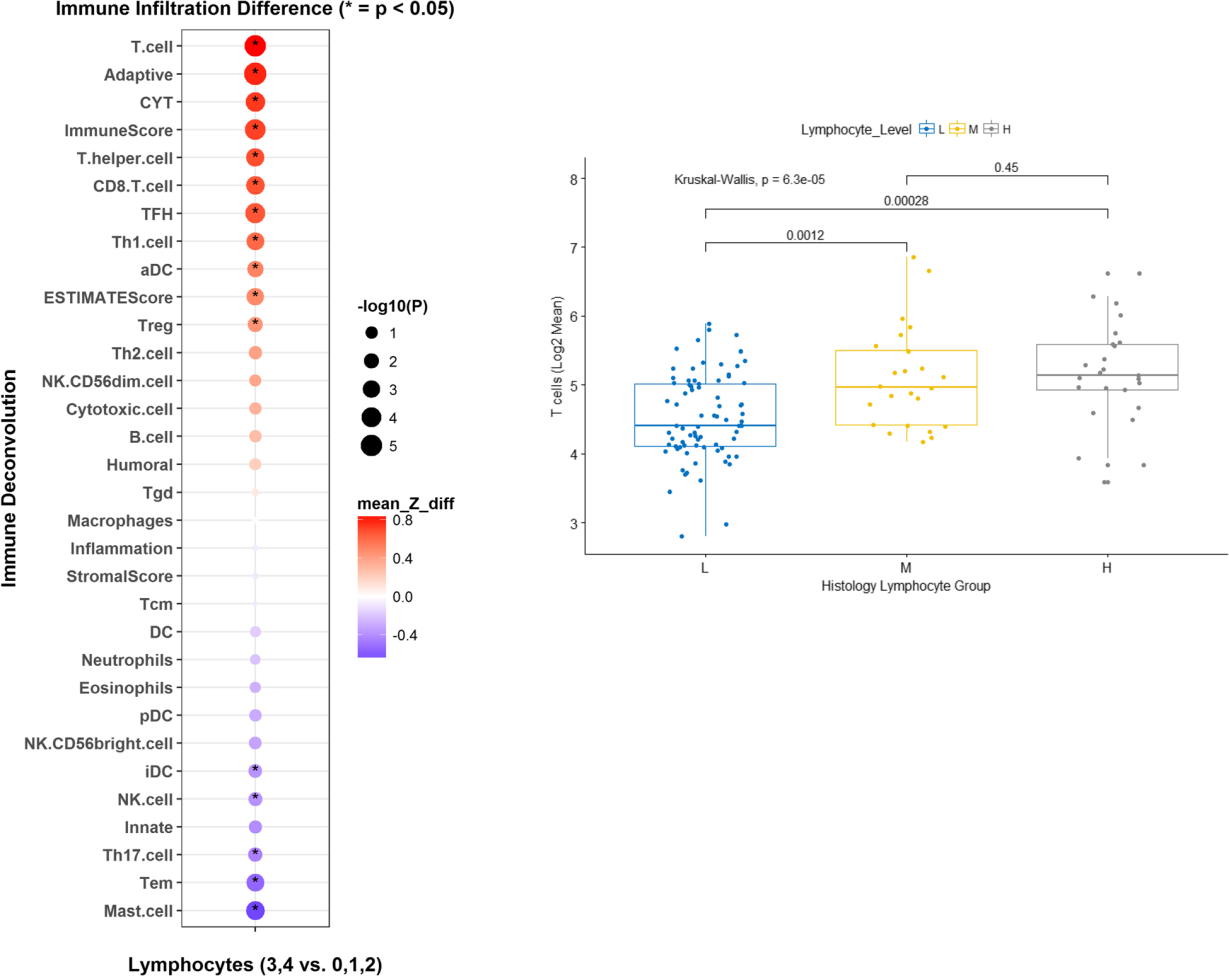
Left: higher lymphocyte infiltration is associated with higher T cells, adaptive immune response, T cells, CYT, ImmuneScore, T helper cells, Tregs, Th1, cytotoxic CD8 T cells. Right: Higher morphological lymphocyte infiltration correlates with higher T cell score based on NanoString gene sets (L = infiltration score 0, 1, 2; M = 3; H = 4)

For morphologic assessment of immune cells in the UAB discovery dataset, we identified 159 patients who met inclusion criteria. In this cohort 33/159 (20.7%) had disease recurrence and 126/159 (79.3%) were free of disease (Fig. 1). We identified 198 patients from the FCCC database, of which 53/198 (26.7%) had disease recurrence and 145/198 (73.3%) were free of disease (Fig. 2). Patient and pathologic characteristics from both datasets are listed in Table 1. The median age in both groups was similar (58–61 years) with a male predominance. The median time to recurrence was 25.8 months (range, 4.2–114.7 mo) in the UAB group, and 26.1 months (2.3–85 mo) in the FCCC group.

**Table 1:** Patient and tumor characteristics

Characteristic	UAB discovery dataset		Fox chase validation dataset	
	Number (*N* = 159), %	Recurrers (*N* = 33), %	Number (*N* = 198), %	Recurrers (*N* = 53), %
Median Age	58	59	61	59
Gender				
Female	60 (37.8)	11 (33.3)	55 (27.7)	13 (24.5)
Male	99 (62.2)	22 (66.7)	143 (72.2)	40 (75.4)
Race				
Caucasian	124 (77.9)	30 (90.9)	190 (96)	51 (96.2)
AA	19 (12.0)	2 (6)	6 (3.0)	2 (3.7)
Other	16 (10.1)	1 (3)	2 (1)	0 (0)
Median Follow-up	48.3 mo (24.5–120.5)	56 mo (25.5–119.7)	70.92 (15.4–126.9)	61.9 mo (16.2–126.9)
Median time to recurrence		25.8 mo (4.2–114.7)		18.4 mo (2.3–85.3)
Pathologic T stage				
T1 / T2 / T3/ T4	62 / 20 / 77 / 0(38.9 / 12.6 / 48.5)	4 / 6 / 23 / 0 (12.1 / 18.1 / 69.7)	89 / 31 / 76 / 2 (45 / 15.6 / 38.3 / 1)	17 / 6 / 28 / 2 (32 / 11.3 / 52.8 / 3.7)
Grade				
1–2	43 (27)	6 (18.1)	58 (29.2)	9 (16.9)
3–4	116 (73)	27 (81.8)	140 (70.7)	44 (83)
Necrosis				
Yes	48 (30.2)	18 (54.5)	61 (30.8)	28 (52.8)
No	111 (69.8)	15 (45.4)	137 (69.1)	25 (47.1)
Lymphocyte infiltration scoring				
0	10 (6.2)	0 (0)	27 (13.6)	5 (9.4)
1	56 (35.2)	7 (21.2)	44 (22.2)	10 (18.9)
2	30 (18.8)	9 (27.2)	48 (24.2)	9 (17)
3	33 (20.7)	7 (21.2)	31 (15.6)	10 (18.9)
4	30 (18.8)	10 (30.3)	48 (24.2)	19 (35.8)

Adequate tumor tissue was available in 132/159 patients for NanoString analysis in the UAB group, of which 24/132 (18%) had disease recurrence. Gene expression data from 5 cases was discarded as they did not meet normalization and quality control requirements (Fig. 1). Thus, NanoString gene expression data was analyzed in a total of 127 patients with 24 recurrences. The patient and tumor characteristics are demonstrated in Table 1. The TCGA dataset comprised of 414 patients with available RNASeq data. After excluding AJCC Stage IV patients, 330 had available recurrence status and of them 69 were recurrent and 261 were non-recurrent.

### Morphologic TILs correlated with T cell gene expression

Deconvolution of the immune cell mRNA gene expression data using NanoString showed that higher morphologically identified TILs correlated with higher Tcell gene expression (*p* = 0.00028 when comparing low (0,1,2) vs high [4] infiltration of T cells) (Fig. 2 right). Additionally higher TILs also correlated with higher T cell, ImmuneScore, Tregs, CYT, Th1, Adaptive immune response, T helper cell and CD8+ T cells (Fig. 2 left). Of note, there was only one gene representing Tregs in the annotated NanoString gene panel (*FOXP3*).

### Recurrence correlated with lower T cells and adaptive immune cells in UAB and TCGA KIRC dataset by deconvolution of mRNA expression data

**Fig. 3:**
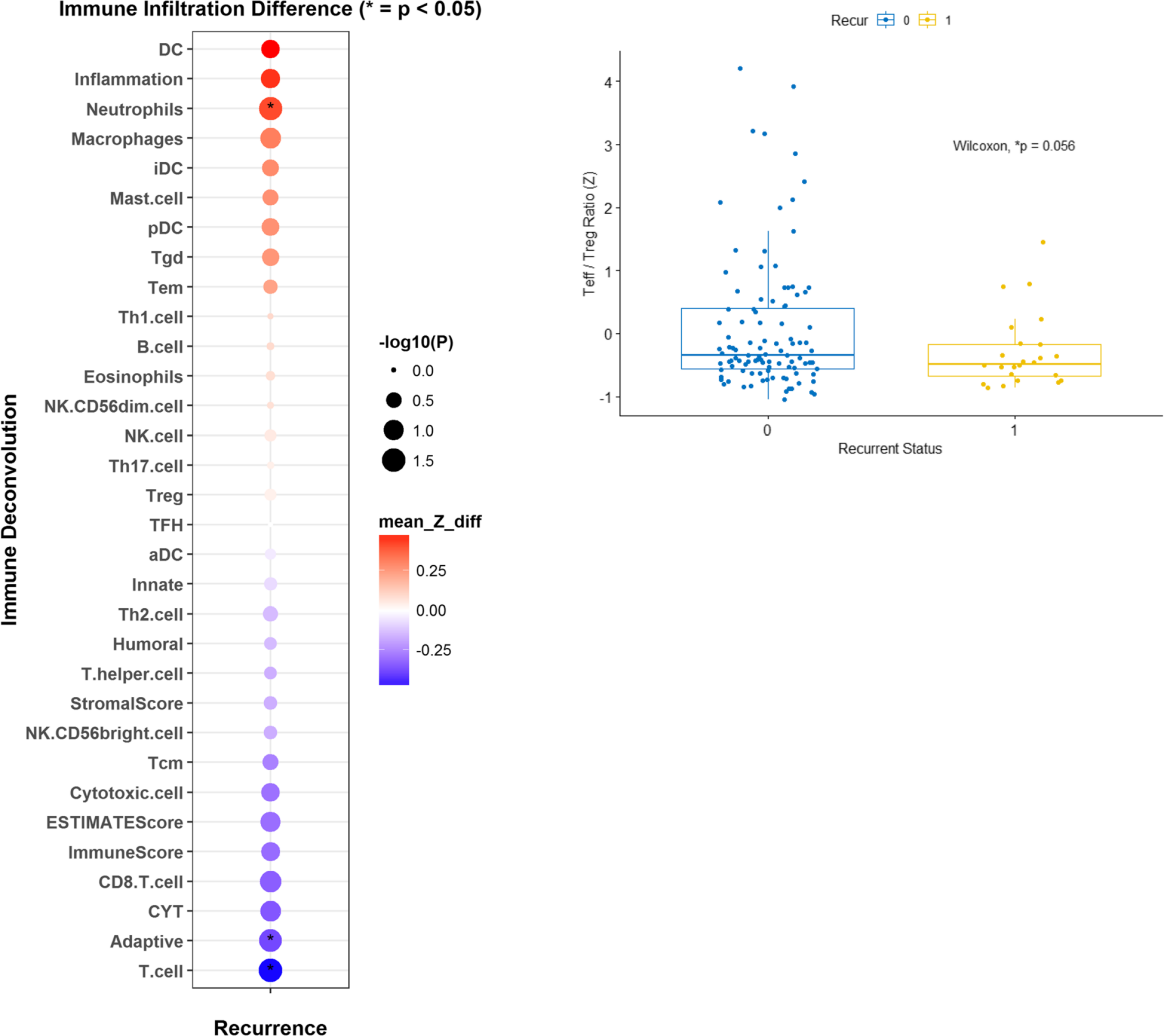
Left: Association of recurrence with immune cell types using NanoString gene sets on UAB dataset using log2mean method. Red represents overexpression and blue represents decreased expression. Recurrence is associated with a significantly increased expression of neutrophils and lower expression of T cells and adaptive immune response. Right: Teff/Treg ratio is associated with lower recurrence with a trend towards significance (* denotes one-sided *p*-value (*p* = 0.056) and the two-sided p-value is 0.11

When correlating mRNA gene expression with recurrence, we found that recurrence was associated with lower T cells (*p* = 0.0295), lower adaptive immune cells (*p* = 0.04) and higher neutrophils (*p* = 0.0377) (Fig. 3). The ratio of Teffector (Teff) /Treg also trended towards a lower rate of recurrence (one-sided *p* = 0.056) (Fig. 3 right). However, when comparing the fold change of mean mRNA expression between recurrence and non-recurrence only 9 genes had a > 1.5 times fold change and false discovery rate (FDR) < 0.3 between recurrence and non-recurrence. (IL8 (CXCL8), NCAM1 (CD56), COL3A1, PPBP (CXCL7) were over-expressed and CX3CL1, CCL4 (LAG1), VCAM1, IL17RB, CXCL14 were under-expressed in recurrence compared to non-recurrence (*p* < 0.05).

**Fig. 4:**
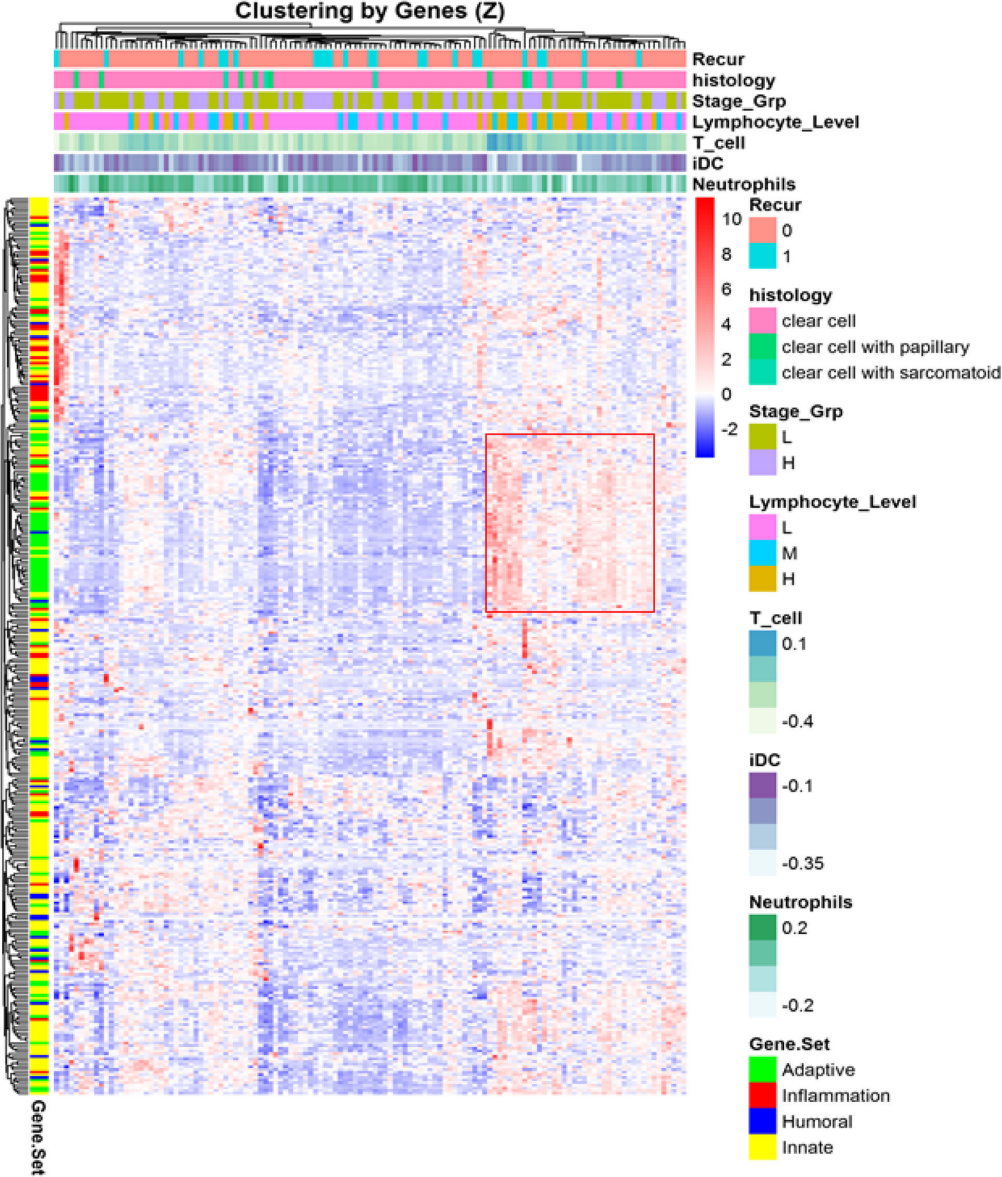
Unsupervised hierarchical clustering of NanoString immune related gene subsets in patients with localized renal cell carcinoma. The highlighted area indicates patients with high morphologic lymphocytes and high expression of adaptive immune genes

In the TCGA dataset, recurrence was significantly associated with higher plasmacytoid dendritic cells (pDC), higher Treg, higher activated dendritic cells (aDC) and higher Th2 cells (*p* < 0.05) (Additional file 1: Figure S1). A higher Teff/Treg ratio was significantly associated with lower recurrence (one-sided *p* = 0.0001) (Additional file 1: Figure S1). Unsupervised hierarchical clustering of NanoString immune response categories in patients did not reveal a clustering of recurrent cases. However, the highlighted area in Fig. 4 indicates a subgroup of patients with high morphologic lymphocytes and high expression of adaptive immune genes.

### Association between morphologic TILs and clinicopathologic characteristics

We focused our analysis on lymphocyte/plasma cells as that was the predominant cell type in ccRCC and other cell types (neutrophils, macrophages) were infrequently observed with low absolute numbers. For the purpose of analysis a score of 0, 1 was considered low and 2, 3, 4 was high. In both the UAB and FCCC groups higher TIL with associated with high grade (> 3) and necrosis (*p* < 0.05) but not high stage (Stage T2 and above) (Table 2). In the UAB group there was significant correlation between high lymphocyte/plasma cell infiltration and higher risk of recurrence (OR 3.08 (1.19, 9.06), *p* = 0.015). Using these cutoffs, TIL score was not statistically significantly associated with recurrence in the FCCC dataset (OR 1.59 (0.77, 3.41), *p* = 0.24) (Table 3). However, when choosing a different cut off (TIL scoring 0, 1, 2 as low and 3, 4 as high), there was a significant correlation in the FCCC group between higher TIL score and higher recurrence (OR 2.29 (1.15, 4.58), *p* = 0.014).

**Table 2:** Association between morphologic TIL and pathologic variables

Characteristic	UAB discovery dataset			FCCC validation dataset		
	Odds ratio	95% CI	Overall *P* value	Odds ratio	95% CI	Overall *P* value
Pathologic stage: Stage> 1 vs 1	1.46	0.5–4.6	> 0.05	1.57	0.9–2.8	> 0.05
Necrosis: Yes vs No	3.73	1.6–9	0.003	2.7	1.3–5.5	0.005
Grade: High (3, 4) vs Low (1, 2)	5.32	1.1–26.3	0.037	3.5	1.9–6.7	0.0001

**Table 3:** Univariate analysis for association of baseline variables with objective tumor recurrence

Characteristic	UAB discovery dataset			FCCC validation dataset		
	Odds ratio	95% CI	Overall *P* value	Odds ratio	95% CI	Overall *P* value
Pathologic stage: Stage> 1 vs 1	6.11	1.5–24.6	0.0015	2.62	1.3–5.3	0.003
Necrosis: Yes vs No	3.8	1.7–8.5	0.001	3.5	1.7–7.2	0.0002
Grade: High (3, 4) vs Low (1, 2)	8.2	2.1–32.2	0.0052	2.48	1.1–6.3	0.02
Lymphocytes/plasma cells: High (2–4) vs Low (0,1)	3.08	1.19–9.06	0.015	1.59	0.77–3.14	0.24
Lymphocytes/plasma cells: High (3, 4) vs Low (0,1, 2)	1.99	0.85–4.72	0.11	2.29	1.15–4.58	0.014

**Fig. 5:**
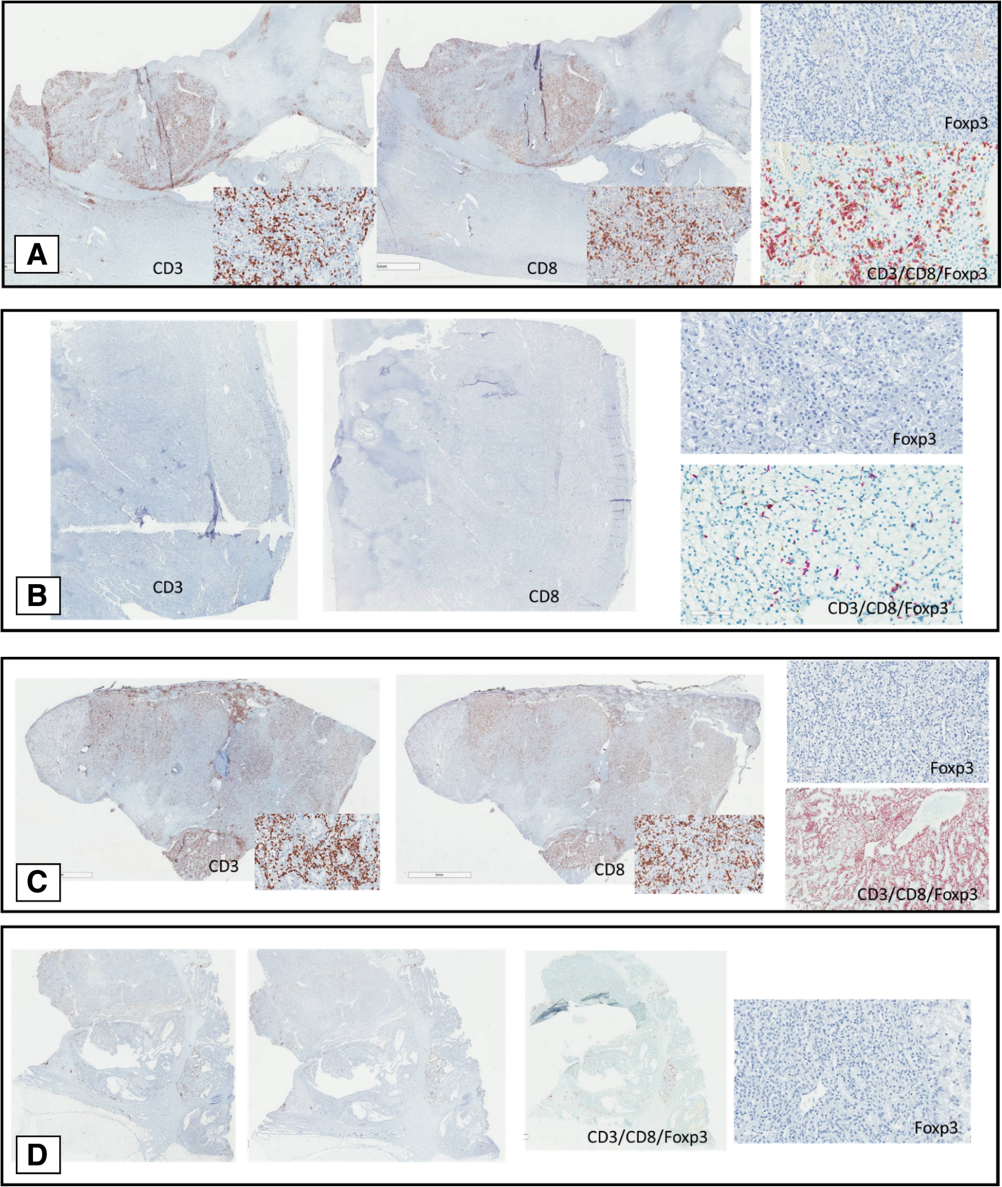
Immunohistochemistry of selected cases with CD3, CD8, Foxp3 and triple staining. **a**. High TILs were identified by morphologic assessment and high T cells were identified by NanoString. IHC confirms presence of high CD3+ and CD8+ T cells. **b**. Low TILs were identified by morphologic assessment and low T cells were identified by NanoString. IHC confirm presence of low CD3+ and CD8+ T cells. **c**. Low TILs by morphologic assessment and high T cells by NanoString were reported. IHC confirmed strong expression of CD3+ and CD8+ T cells. **d**. High TILs by morphologic assessment but low T cells by NanoString were reported. IHC favored NanoString findings with low CD3+ and CD8+ T cells. Minimal Foxp3+ T cell expression noted in all cases

To analyze cases where there was discordance between morphologic TIL scoring and NanoString T cell expression, we stained 11 cases with anti-CD3, CD8 and Foxp3 antibodies. We selected cases falling into each of the following categories: high morphologic TILs/high NanoString CD8 expression; high TILs/low CD8; low TILs/high CD8 and low TILs/low CD8. The level of expression consistent correlated with NanoString T cell expression but not morphologic TIL scoring. In cases with high NanoString T cell expression, strong expression of CD3+ and CD8+ T cells was noted on IHC. Likewise, low NanoString T cell expression correlated with minimal CD3+ and CD8+ T cells by IHC. (Fig. 5 and Additional file 2: Table S1). Foxp3 expression was low across all cases, consistent with NanoString results.

We suspect that the lack of consistent association between morphologic TILs and recurrence is likely related to the subjective nature of scoring. Also, since the morphologic method clumps all TILs and does not distinguish between T cell subtypes, which are clearly significant based on the NanoString data, a more robust scoring method may need to be developed to determine prognosis.

## Discussion

The purpose of the study was to identify an immune gene expression signature to predict recurrence in patients with localized ccRCC who undergo nephrectomy. Deconvolution of NanoString data to identify cell types showed that lower T cell and adaptive immune gene expression correlated with recurrence. Additionally, a higher Teff/Treg ratio was associated with lower recurrence, which was validated in the TCGA dataset. Of note, gene expression of specific cell types eg. Th1, Th2, CD8 T cells was not different between recurrent and non-recurrent tumors. There was a strong correlation between morphologically identified TILs and gene expression for T cells, CD8+ T cells, Th1 cells, Tregs and cytotoxic T cells, suggesting that these are the most abundant cell types in the tumor microenvironment in localized RCC. The NanoString results were confirmed by IHC results where high CD3+ and CD8+ expression correlated with high T cell gene expression by NanoString.

We were surprised to find that between recurrers and non-recurrers only 9 out of 730 immune related genes were differentially expressed significantly with a > 1.5 times fold change and FDR < 0.3. An important observation here is that the overall immune related gene expression in the primary tumor at the time of nephrectomy may not be sufficient to predict recurrence, and that modulation of the immune microenvironment may be occurring during the time period between nephrectomy and recurrence. Only a fraction of the recurrent tumors had a strong expression of *IDO1, CTLA4, PDL1, ICOS* and *TIGIT*, which are associated with negative immune regulation and the expression of these genes did not differ significantly between recurrent and non-recurrent tumors. Giraldo et al. reported that the immune-regulated phenotype in localized RCC characterized by polyclonal/poorly cytotoxic CD8^+^PD-1^+^Tim-3^+^Lag-3^+^ TILs and CD4^+^ICOS^+^ cells with a Treg phenotype (CD25^+^CD127^−^Foxp3^+^/Helios^+^GITR^+^) had high risk of recurrence [16]. In our study, this signature did not distinguish tumors that recurred from non-recurrers.

Our study has several caveats that need to be considered. In the breast cancer literature, a standardized methodology for morphological evaluation of TILs has been provided by the International TILs working group and has been found to have a prognostic and predictive role [17–19]. In contrast to breast cancer, ccRCC has much higher lymphocyte infiltration in the tumor center as well at the tumor margin and so in our study intra-tumoral as well as TILs at the tumor margin were quantified, unlike the definition of the breast cancer TIL working group. The significance of including immune cells in the tumor center vs tumor margin is unclear at this time in ccRCC.

Recent technological developments in single cell cytometry and tissue imaging may allow more accurate assessment of individual immune cells separated from tumor cells [20]. However, to develop a tool to predict recurrence that can be applied in the clinic, it is worth investigating FFPE tissue samples that are easily available. Additionally the spatial architecture and arrangement of TILs may play a prognostic role in RCC as has been noted in other cancers [21]. In the future we hope to use descriptors that capture density and spatial co-localization of TILs and tumor cells across digital images to better characterize TILs. Estimation of tumor microenvironment composition using gene expression data is not novel and several methods have been previously used [22–25]. We used the gene annotation data provided by NanoString, which includes 24 immune cell type gene signatures and 4 immune response categories. While the limited panel was adequate to decipher the immune populations, especially lymphocyte populations (based on Fig. 2 left), most previous studies have utilized bulk microarray/RNASeq data for immune deconvolution [22, 23]. Finally, tumor heterogeneity is an issue that is hard to overcome in transcriptomic studies. However, our morphologic review of every section of the tumor, and selection of blocks with most TILs for NanoString analysis may help partially address this issue.

Several ongoing trials are studying (neo) adjuvant immunotherapy for ccRCC and results are awaited (NCT03341845, NCT03142334​, NCT03024996) [26]. It will be interesting to see if modulating the immune microenvironment in the perioperative setting will decrease recurrence in ccRCC. This question is especially important given the finding of our study that there is a fairly homogenous immune gene expression profile in primary ccRCC tumors at the time of nephrectomy.

## Conclusion

The standard of care for localized RCC post nephrectomy is observation. As several clinical trials are investigating the role of perioperative immunotherapy to decrease recurrence of localized RCC, characterizing the role of immune cells infiltrating primary RCC tumors is vital. The immune composition of localized RCC comprises mainly of lymphocytes with infrequent Foxp3+ Tregs. We found that the presence of higher T cell infiltration correlated with lower chances of recurrence. The presence of an adaptive immune response gene signature was also only identified in few tumors and whether perioperative immunotherapy may enable activation of existing immune infiltrate and reduce recurrence in localized RCC is a subject of ongoing investigation.

## Additional files

Additional file 1: Association of recurrence with immune cell types using NanoString gene sets on TCGA KIRC dataset. (DOCX 105 kb)

Additional file 2: CD8 IHC data. (DOCX 12 kb)

## Data Availability

The datasets used and/or analyzed during the current study are available from the corresponding author on reasonable request.
