# Correction to: Prognostic impact of immune gene expression signature and tumor infiltrating immune cells in localized clear cell renal cell carcinoma

**DOI:** 10.1186/s40425-019-0735-5

**Published:** 2019-10-22

**Authors:** Pooja Ghatalia, Jennifer Gordetsky, Fengshen Kuo, Essel Dulaimi, Kathy Q. Cai, Karthik Devarajan, Sejong Bae, Gurudatta Naik, Timothy A. Chan, Robert Uzzo, A. Ari Hakimi, Guru Sonpavde, Elizabeth Plimack

**Affiliations:** 1 Fox Chase Cancer Center, 333 Cottman Ave, Philadelphia, PA 19111 USA; 2 University of Alabama at Birmingham, 1802 6th Ave S, Birmingham, AL 35233 USA; 3 Memorial Sloan Kettering Cancer Center, 1275 York Avenue, New York, NY 10065 USA; 4 Dana Farber Cancer Institute, 450 Brookline Ave, Boston, MA 02215 USA

**Correction to: J Immunother Cancer**

**https://doi.org/10.1186/s40425-019-0621-1**

Following publication of the original article [1], the author reported that the current funding section “Kidney Cancer Association Young Investigator Grant provided funding for this project” should be replaced with “Kidney Cancer Association Young Investigator Grant and Bucks County Board provided funding for this project.”
