# Supplementary material for: Prognostic impact of immune gene expression signature and tumor infiltrating immune cells in localized clear cell renal cell carcinoma
Source: J Immunother Cancer. 2019 May 28;7:139. doi: 10.1186/s40425-019-0621-1 (PMC6540413; doi:10.1186/s40425-019-0621-1)
Supplement: Supplementary file 1 — Association of recurrence with immune cell types using NanoString gene sets on TCGA KIRC dataset. (DOCX 105 kb) [file 40425_2019_621_MOESM1_ESM.docx]

**
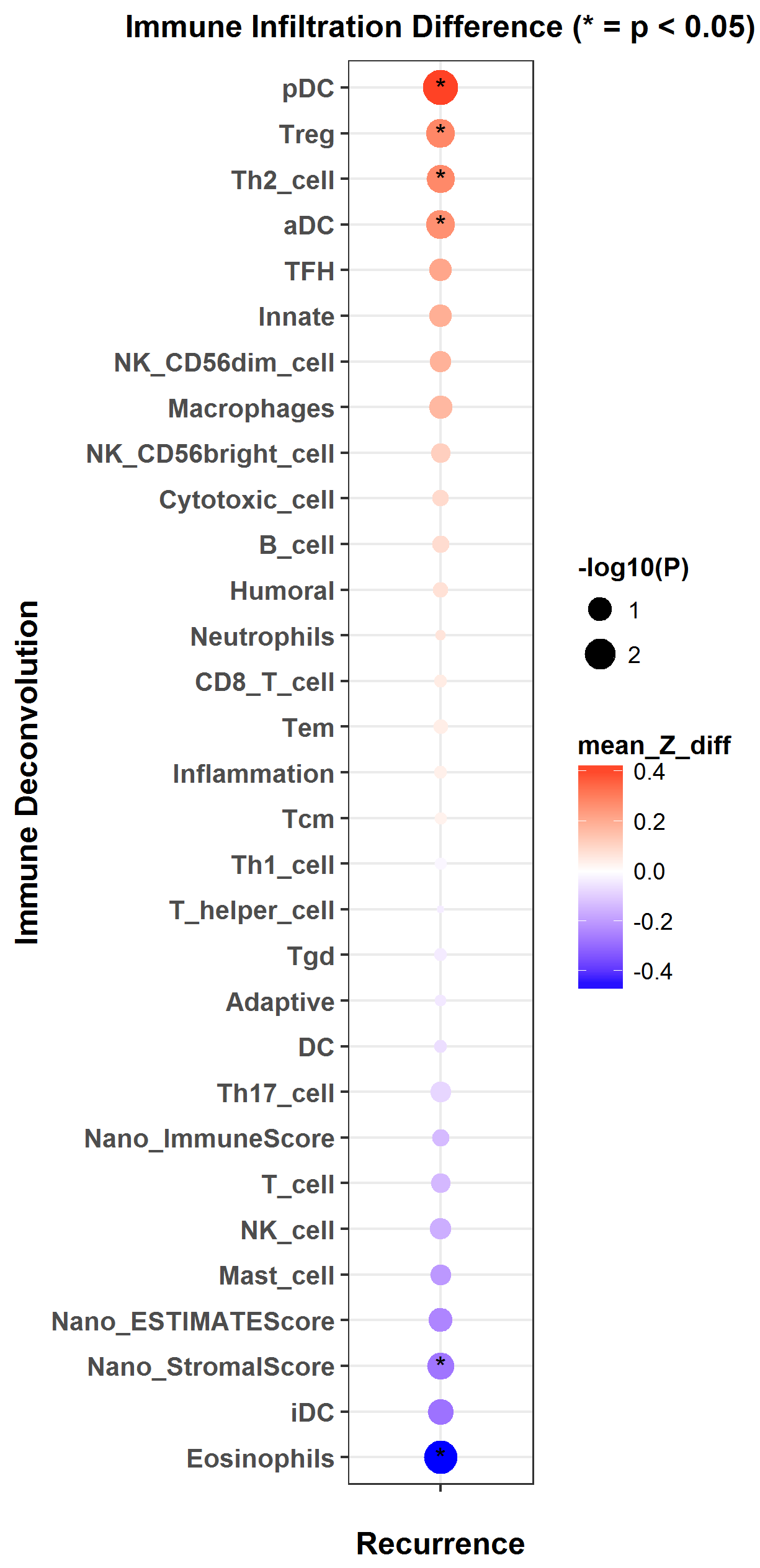
**’
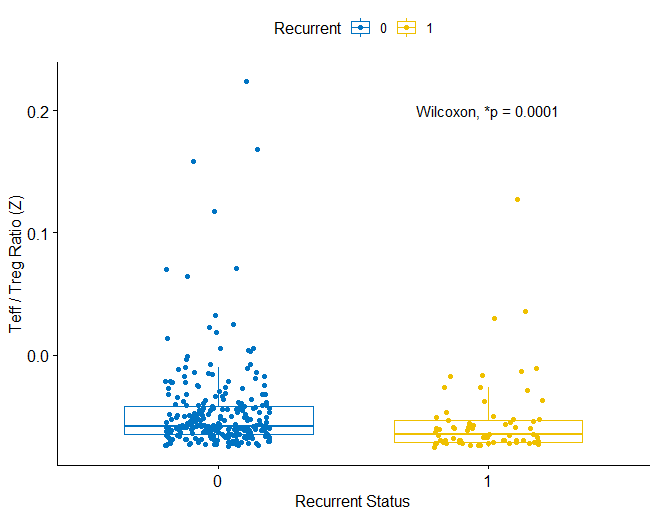


**Supplemental fig1: Left:** Association of recurrence with immune cell types using NanoString gene sets on TCGA KIRC dataset using log2 mean method. Red denotes overexpression and blue denotes decreased expression. Recurrence is significantly associated with increased expression of Tregs, Th2 cells, activated and plasmacytoid dendritic cells and decreased expression of eosinophils. **Right:** Significant association between Teff/Treg ratio with lower recurrence.* denotes one-sided p-value (p=0.0001), two-sided p-value is 0.002
