# Supplementary material for: Prognostic impact of immune gene expression signature and tumor infiltrating immune cells in localized clear cell renal cell carcinoma
Source: J Immunother Cancer. 2019 May 28;7:139. doi: 10.1186/s40425-019-0621-1 (PMC6540413; doi:10.1186/s40425-019-0621-1)
Supplement: Supplementary file 2 — CD8 IHC data. (DOCX 12 kb) [file 40425_2019_621_MOESM2_ESM.docx]

| Case number | Morphologic TIL score | NanoString CD8 score | IHC CD8 score | IHC Foxp3 score |
| --- | --- | --- | --- | --- |
| Cases with high morphologic TILs and high NanoString CD8 score | | | | |
| 1 | 4 | 6.28 | ++++ | - |
| 2 | 4 | 6.01 | +++ | - |
| 3 | 4 | 6.62 | ++++ | - |
| Cases with low morphologic TILs and high NanoString CD8 score | | | | |
| 4 | 1 | 5.06 | ++ | + |
| 5 | 1 | 5.52 | +++ | - |
| 6 | 1 | 5.88 | +++ | - |
| Cases with high morphologic TILs and low NanoString CD8 score | | | | |
| 7 | 4 | 3.59 | + | - |
| 8 | 4 | 3.84 | - | - |
| 9 | 4 | 3.94 | + | + |
| Cases with low morphologic TILs and low NanoString CD8 score | | | | |
| 10 | 1 | 2.8 | - | - |
| 11 | 1 | 3.44 | - | - |

Supplemental Table 1: Random cases with concordance and discordance between morphologic TIL scoring and NanoString CD8 score were selected for IHC of CD8+ cells. As noted above, in cases with discordance between morphologic and NanoString scoring, IHC correlates with NanoString CD8 scoring.
